# Accelerated Development of Rod Photoreceptors in Retinal Organoids Derived from Human Pluripotent Stem Cells by Supplementation with 9-*cis* Retinal

**DOI:** 10.1016/j.xpro.2020.100033

**Published:** 2020-06-03

**Authors:** Ryan A. Kelley, Holly Y. Chen, Anand Swaroop, Tiansen Li

**Affiliations:** 1 Neurobiology, Neurodegeneration & Repair Laboratory, National Eye Institute, National Institutes of Health, Bethesda, MD, USA

## Abstract

Human pluripotent stem cells (PSCs) can be differentiated into retinal organoids with proper neural layer organization, yet the protocols are technically challenging and time consuming. We have modified a widely used differentiation protocol by switching all-*trans* retinoic acid with 9-*cis* retinal to accelerate photoreceptor differentiation and improve morphogenesis. In this report, we provide a detailed and improved protocol to generate retinal organoids from human pluripotent stem cells.

For complete details on the use and execution of this protocol, please refer to Kaya et al. (2019).

## Highlights

• Detailed protocol of retina organoid differentiation from human pluripotent stem cells
• Supplementation with 9-*cis* retinal improves retina organoid development
• 9-*cis* retinal accelerates photoreceptor maturation in retina organoids

## Graphical Abstract

**Figure:**
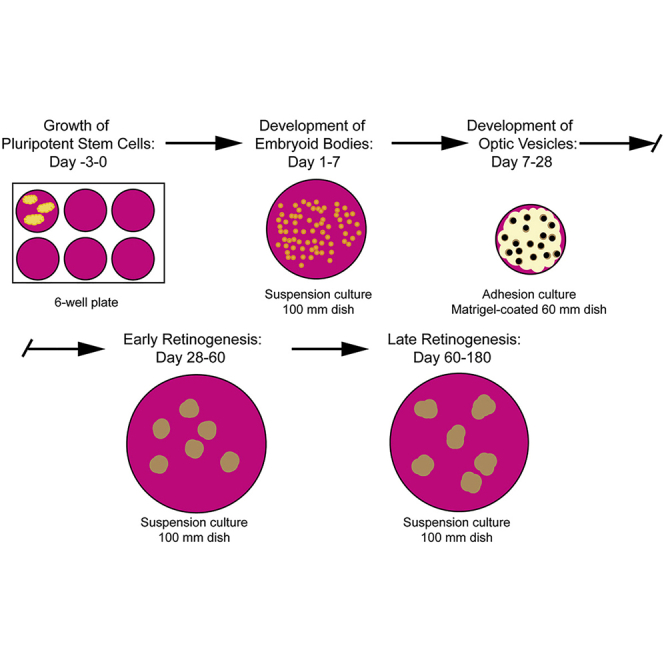

## BEFORE YOU BEGIN

**CRITICAL:** All procedures should be conducted under a Class II Biological Safety Cabinet (BSC) to maintain sterility.

### Reconstitution of Reagents

**Timing: 2.5–3.0 h**

1. 10 mM Rock Inhibitor (1,000X)
  a. Add 3.12 mL of ultra-pure water to 10 mg Rock Inhibitor (Y-27632).
  b. Aliquot 100 μL into 1.5 mL microcentrifuge tubes
  c. Store aliquots at -80°C until use. Once thawed, keep the tube at 4°C to avoid freeze-and-thaw cycles.
2. 20 mg/mL Heparin (10,000X)
  a. Add 100 mL of ultra-pure water to 20 mg heparin.
  b. Aliquot 10 mL into 15 mL falcon tubes
  c. Wrap caps in parafilm and store at 4°C until use.
3. 100 mM Taurine (1,000X)
  a. Add 40 mL ultra-pure water to 500 mg taurine. Shake for ∼1 hour at 20-22°C to dissolve all taurine.
  b. Filter taurine solution with 0.22 μm filter.
  c. Wrap caps in parafilm and store at 4°C until use.
4. 10 μg/mL insulin-like growth factor 1 (IGF1, 500x)
  a. Add 1 mL ultra-pure water to 100 μg IGF1 powder purchased from a commercial supplier. Invert the tube several times to put all powder into solution. Spin down briefly.
  b. Aliquot 900 μL DMEM:F12 into ten 1.5 mL tubes.
  c. Add 100 μL of the 100 μg/mL IGF1 solution to each DMEM:F12 tube. Invert the tube multiple times to mix solution.
  d. Spin down briefly. Store aliquots at -20°C until use.

**CRITICAL:**

• IGF1 is stable at -20°C for approximately half a year. After thawing, store at 4°C for no more than 1 week.
5. 10 mM 9-*cis* retinal (10,000X or 20,000X)
  a. Gently tap 25 mg 9-*cis* retinal powder to avoid powder sticking to the cap. Add 1.25 mL DMSO to the powder. Mix well by pipetting up and down several times.
  b. Aliquot 100 μL of the 70 mM stock into 1.5 mL amber microcentrifuge tubes.
  c. Store stock solution at -80°C.
  d. Add 10 μL stock solution to 60 μL DMSO to prepare a 10 mM working solution.
  e. Store working solution at -20°C until use.

**CRITICAL:**

• Handle 9-*cis* retinal under dim red light.
• Stock solution can be stored at -80°C for approximately one year. The 10 mM working solution can be stored at -20°C for approximately half a year.
6. 10 mM all-*trans* retinoic acid (10,000X or 20,000X)
  a. Gently tap 50 mg all-*trans* retinoic acid powder to avoid powder sticking to the cap. Add 1.67 mL DMSO to the tube. Mix well by pipetting up and down several times.
  b. Aliquot 100 μL of the 100 mM stock into 1.5 mL amber microcentrifuge tubes.
  c. Store stock solution at -80°C.
  d. Add 10 μL stock solution to 90 μL DMSO to prepare a 10 mM working solution.
  e. Store working solution at -20°C until use.

**CRITICAL:**

• Stock solution can be stored at -80°C for approximately one year. The 10 mM working solution can be stored at -20°C for approximately half a year.

### Preparation of Tissue Culture Ware

**Timing: 1–2 h**

7. Matrigel coating (hESC-qualified)
  a. Thaw a bottle of Matrigel in a covered Styrofoam box full of ice. Store the box at 4°C overnight (at least 12 hours). Also freeze 1.5 mL tubes and 1 mL pipette tips at -20°C
  b. The next day, place tubes and Matrigel on ice in a Styrofoam container.
  c. Gently invert Matrigel bottle until suspension is homogenous.
  d. Quickly make 240 μL aliquots (9.5 mg/mL) with the chilled tubes and pipette tips, all on ice.
  e. Store the aliquots at -80° C.
  f. Add 1 aliquot of Matrigel to 25 mL of cold DMEM:F12 on ice and mix well.
  g. Add 1 mL of the diluted Matrigel to each well of 4 x 6-well tissue culture plates using pre-chilled pipette tips
  h. Wrap plates individually in parafilm and cover all in aluminum foil to protect from light.
  i. Plates can be stored at 4°C for up to 2 weeks
8. Matrigel coating (growth factor reduced)

Aliquots of 240 μL Matrigel (9.5 mg/mL) are made in 1.5 mL tubes and frozen at -80°C until use. However, the concentration of Matrigel can vary across lots. The appropriate volume should be adjusted according to the concentration of each lot found at Corning Life Science’s website in the “download certificates” section.

a. Thaw a bottle of Matrigel in a covered Styrofoam box full of ice. Store the box at 4°C overnight (at least 12 hours). Also freeze 1.5 mL tubes and 1 mL pipette tips at -20°C
b. The next day, place tubes and Matrigel on ice in a Styrofoam container.
c. Gently invert Matrigel bottle a few times until suspension is homogenous
d. Quickly make aliquots in the chilled tubes and with chilled pipette tips, all on ice.
e. Store the aliquots at -80°C.
f. Add 1 aliquot of Matrigel to 18 mL of cold DMEM:F12 on ice.
g. Add 3 mL to each of 6 x 60 mm tissue culture dishes using pre-chilled pipette tips
h. Wrap dishes individually in parafilm and cover in aluminum foil to protect from light.
i. Plates can be stored at 4°C for up to 2 weeks
9. Poly 2-hydroxyethyl methacrylate (polyHEMA) coating
  a. Prepare a 2 mg/mL solution of polyHEMA in 95% ethanol. Incubate the solution at 65°C until completely dissolved. Resulting stock can be stored at 20-22°C.
  b. Rinse non-treated 60mm and 100mm petri dishes in polyHEMA solution. Unused solution can be added back to the stock.
  c. Sterilize overnight (at least 12 hours) under UV light in the hood.
  d. Wrap individual plates in parafilm and store at 22-25°C until use.

## KEY RESOURCES TABLE

**Table:** 

REAGENT or RESOURCE	SOURCE	IDENTIFIER
**Chemicals, Peptides, and Recombinant Proteins**		

Rock inhibitor (Y-27632)	Tocris	1254
Heparin	Sigma	H3393
Taurine	MilliporeSigma	T5691
IGF1 Recombinant Human Protein	ThermoFisher	PHG0071
9-*cis* retinal	MilliporeSigma	R5754
All-*trans* retinoic acid	MilliporeSigma	R2625
Matrigel® Growth Factor Reduced (GFR) Basement Membrane Matrix	Corning	354230
Matrigel® hESC-qualified Basement Membrane Matrix	Corning	354277
Essential 8 media	ThermoFisher	15169-01
DMEM	ThermoFisher	11995073
F12	ThermoFisher	11765054
Essential 8 supplement	ThermoFisher	15171-01
B-27® Supplement (50X), minus vitamin A	ThermoFisher	12587010
N-2 Supplement (100X)	ThermoFisher	17502048
2-Mercaptoethanol	ThermoFisher	21985023
MEM Non-essential Amino Acid Solution (100X)	MilliporeSigma	M7145
GlutaMAX™ Supplement	ThermoFisher	35050061
Fetal Bovine Serum (freezing media)	ThermoFisher	16000069
Antibiotic-Antimycotic	Gibco	15240096
DMSO	Sigma-Aldrich	D4540
EDTA solution	Corning	46-034CI
Poly(2-hydroxyethyl methacrylate)	MilliporeSigma	P3932

**Experimental Models: Cell Lines**		

H9 human embryonic stem cells	WiCELL	WA09

**Other**		

60 mm non-treated plates	FisherBrand	FB0875713A
100 mm non-treated plates	FisherBrand	FB0875715
6-well (TC-treated) plates	Corning	353046
60 mm (TC-treated) plates	Corning	430196
Polystyrene serological pipettes	Corning	4490, 4489, 4488, 4487, 4486
15 mL conical centrifuge tubes	Corning	430766
50 mL conical centrifuge tubes	Corning	430290
1.5 mL clear and amber microcentrifuge tubes	USA scientific	1615-5500, 1615-5507
0.22 μm pore size and low protein binding vacuum filter	VWR	514-0332
Pipette tips	Eppendorf	022491253, 022491296, 022491211

## MATERIALS AND EQUIPMENT

• Biological safety cabinet Class II
• -20°C and -80°C freezers
• Liquid nitrogen storage dewar
• Tri-gas CO_2_ incubator
• Water bath
• Centrifuge
• Red light head lamp

### Reconstitution of Media

All media and reagents are filtered through 0.22 μm low protein binding polystyrene membranes, except E8 media.

All media (without N2 or B27 supplements) can be used for up to one month when stored at 4°C, except E8 media.

**CRITICAL:**

• All reagents are warmed at 20-22°C.
• B27 minus Vitamin A and N2 supplements are added after filtration, immediately before adding to culture. DO NOT filter the supplements. Once the supplements are added, the medium should be used within one week.

**Table:** Essential 8 Medium (E8)

Ingredient	Volume	Final Concentration
Essential 8 basal medium	500 mL	-------
Essential 8 supplement	10 mL	2%

**CRITICAL:**

• Due to the instability of fibroblast growth factor 2 (FGF2), reconstituted E8 media should be used within 2 weeks. It is recommended that 50 mL aliquots of reconstituted media be made, and one tube used each time to maintain FGF2 activity.

**Table:** 0.5 mM EDTA Solution

Ingredient	Volume	Final Concentration
0.5 M EDTA	250 μL	0.5 mM
Phosphate buffered saline	250 mL	----

***Note:*** Store at 4°C for no longer than 1 month.

**Table:** Neural Induction Media (NIM)

Ingredient	Volume	Final concentration
DMEM	250 mL	-------
F12	250 mL	
Non-essential amino acids	5 mL	1%
Heparin	50 μL	2 μg/mL
N2 (100x) (added fresh)	5 mL	1%

***Note:*** N2 added fresh before use. Store at 4°C for no longer than 1 month.

**Table:** Freezing Medium

Ingredient	Volume	Final Concentration
FBS	9 mL	90%
Dimethyl sulfoxide (DMSO)	1 mL	10%

***Note:*** Prepared fresh before use. Store at 4°C for at least 5 minutes after reconstitution as mixing is exothermic.

**Table:** 3:1 DMEM:F12 Media (3:1)

Ingredient	Volume	Final Concentration
DMEM	375 mL	-------
F12	125 mL	-------
Non-essential amino acids (100X)	5 mL	1%
Antibiotic Antimycotic (100X)	5 mL	1%

***Note:*** B27 is added fresh before use. Store at 4°C for no longer than 1 month.

**Table:** 3:1 DMEM:F12 + 10% FBS Media (3:1 +FBS)

Ingredient	Volume	Final Concentration
DMEM	337.5 mL	-------
F12	112.5 mL	-------
Certified FBS	50 mL	10%
GlutaMAX (100X)	5 mL	1%
Non-essential amino acids (100X)	5 mL	1%
Antibiotic Antimycotic (100X)	5 mL	1%

***Note:*** B27 or N2 added fresh before use. Store at 4°C for no longer than 1 month.

## STEP-BY-STEP METHOD DETAILS

This protocol uses human embryonic stem cells up to passages (P) 85 and human induced pluripotent stem cells between P15 and P25. Later passages can show reduction in efficiency. The protocol described here is for PSC maintenance in 6-well plates and differentiation in 100 mm dishes. Please adjust the amount of media and reagents accordingly if culture wares of different sizes are used.

### Thaw Cells

**Timing: 1.5 h**

1. Remove parafilm and warm up a Matrigel-coated 6-well plate in 37°C incubator for at least 1 hour.
2. Thaw a frozen vial of human PSC in 37°C water bath until a bit of ice is remaining.
3. Dissolve the remaining ice by pipetting up and down, gently, using wide-bore tips.
4. Gently transfer the cells to a new 15 mL centrifuge tube.
5. Slowly add 10 mL room temperature (20-22°C) E8 media to the cells.
6. Invert tube several times to mix cells in media.
7. Centrifuge the cells at 200 g for 3 minutes.
8. Remove the supernatant and loosen cell pellet by tapping bottom of tube until the pellet dissociates.
9. Resuspend the pellet in 1 mL E8 media with 10 μM rock inhibitor (RI, stock 1:1000).
10. Add cells to one well of a 6-well Matrigel-coated plate.
11. Add 1 mL E8 media with 10 μM RI to the tube to resuspend remaining cells and transfer to the 6-well plate.
12. Shake plate to evenly distribute cells and move plate to the hypoxia incubator: 5% O_2_ at 37°C.

**CRITICAL:**

• Human pluripotent stem cells maintain high viability in small aggregates. Use wide-bore tips and gentle pipetting to avoid dissociating the clumps into single cells.
• At least three passages are needed after cell revival for efficient differentiation.
• Split cells when they reach 60%-80% confluency.

### Day 0: Split Cells

**Timing: 1.5 h**

13. Remove parafilm and warm a Matrigel-coated 6-well plate in 37°C incubator for at least 1 hr.
14. Remove all media from one well containing adherent PSCs.
15. Rinse well twice with 1 mL 0.5 mM EDTA/PBS solution (20-22°C) to chelate Mg++ and Ca++.
16. Incubate cells with 1 mL EDTA/PBS solution for 3-5 minutes.
17. During incubation, remove media from a new well and add 2 mL E8 media with 10 μM RI.
18. Remove EDTA/PBS solution from well and add 1 mL E8 media with 10 μM RI. Dissociate cells by gently pipetting up and down three times, covering the entirety of the well.
19. Check the cells under a microscope. Pipette additional times if large clumps are still observed.

**CRITICAL:**

**Figure 1:**
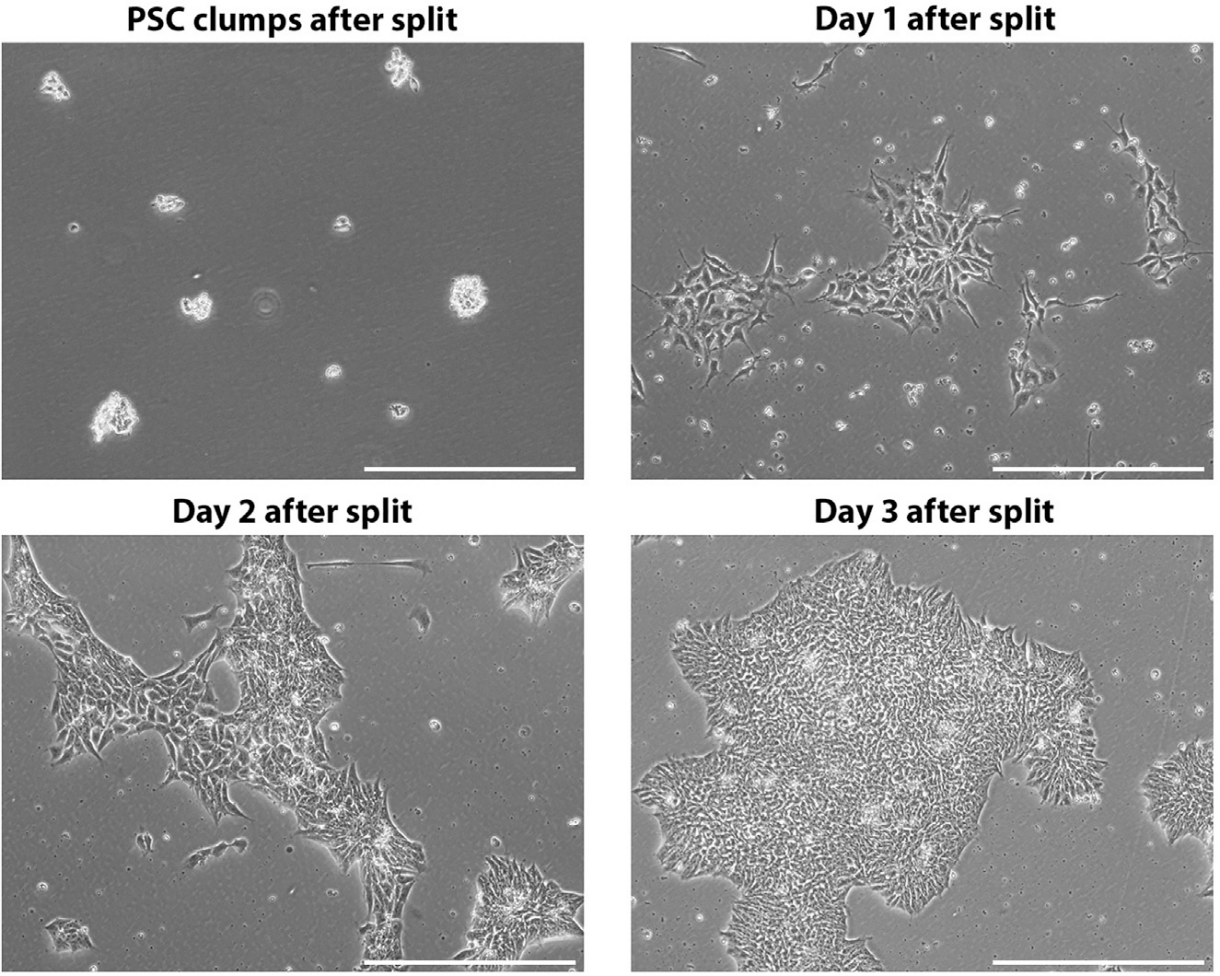
Morphology of Growing Pluripotent Stem Cells Representative bright field images of cells are shown. Immediately after the split (top, left), day 1 after split (top, right), day 2 after split (bottom, left), and day 3 after split (bottom, right). Scale bars=400 μm.

• Avoid dissociating clumps into single cells. See Figure 1 for optimal aggregate size.

20. Transfer 1/6 – 1/12 of the cell suspension to each new well (desired number depends on the dilution factor). Label the new well with name of cell line, date and passage number.

**CRITICAL:**

• The split ratio depends on confluency of the cells. Please refer to Figure 1 for optimal plating density for 3-day culture.
• Plating at too high of a density can result in spontaneous differentiation, while too low can lead to genome instability.

***Optional:***

• The remaining cell suspension can be centrifuged at 200 g for 3 minutes and resuspended in freezing media for storage.
• One well can be resuspended in 4 mL freezing media and made into 1 mL aliquots.
• Add freezing media slowly with constant shaking to facilitate gradual changes in osmolarity.
21. Transfer the remaining cell suspension to a 100 mm polyHEMA-coated dish using a wide-bore tip. Rinse the well with 8 mL E8 media with 10 μM RI and transfer to the plate. Label plate with name of cell line, date and passage number.
22. Add 2 mL E8 media with 10 μM RI to the original well and return it to incubator for backup.
23. Shake the 6-well plate and 100 mm dish to evenly distribute cell suspension and place in hypoxia incubator with 5% O_2_ at 37°C.
24. Add 3 mL E8 media without RI on day 1 and 4 mL on day 2. Cells are ready to split when proper confluency has been achieved (60-80%), when colonies have smooth edges and a high nuclei-to-cytoplasm ratio (Figure 1).

### Day 1: Addition of NIM

**Timing: 5–10 min**

25. Add 4 mL of room temperature (20-22°C) NIM to the 100 mm dish.
26. Shake to distribute cells evenly and return dish to hypoxia incubator.

### Day 2: Addition of NIM

**Timing: 5–10 min**

27. Slowly tilt dish and allow embryoid bodies (EBs) to sink to the bottom. Remove 6 mL E8:NIM media
28. Add 6 mL NIM to the culture at 20-22°C.
29. Shake to distribute EBs evenly and return dish to hypoxia incubator.

### Day 3: Exchange with NIM

**Timing: 10–15 min**

30. Slowly tilt the dish and allow EBs to sink to the bottom. Remove 10 mL E8:NIM media. Leave ∼2 ml media to avoid desiccation.
31. Add 10 mL NIM and shake plate to mix media.
32. Slowly tilt dish to allow EBs to sink to the bottom. Remove 10 mL NIM media.
33. Add 10 mL NIM and shake plate to mix media.
34. Move dish to normoxia incubator.

### Day 7: Transfer to Matrigel-Coated Culture Ware

**Timing: 1 h**

35. Remove parafilm and place Matrigel-coated 60 mm dish (growth factor reduced) in normoxia incubator for at least 1 hr to warm up.
36. Under a microscope in the BSC, remove EBs that are larger than ∼0.25 mm.
37. Remove media from 60 mm dish.
38. Transfer remaining EBs from 100 mm dish to the Matrigel-coated 60 mm.
39. Slowly tilt the 60 mm dish and allow EB to sink to the bottom. Remove ∼10 mL NIM media.
40. Rinse 100 mm dish with 6 mL NIM and transfer the media to the 60 mm dish.
41. Shake to distribute EBs evenly and return the 60 mm dish to normoxia.

### Day 8-15: NIM Media Change

**Timing: 5–10 min**

42. Exchange 8 mL NIM media whenever culture media turns yellow.

### Day 16-28: 3:1 Media Exchange

**Timing: 5–10 min**

43. Remove 8 mL NIM media.
44. Add 8 mL 3:1 media with 2% B27 (stock 1:50).
45. Change media every day.
46. Optic vesicles can be observed between day 16-27.

### Day 28: Dissection of Optic Vesicles

**Timing: 1–1.5 h**

**Figure 2:**
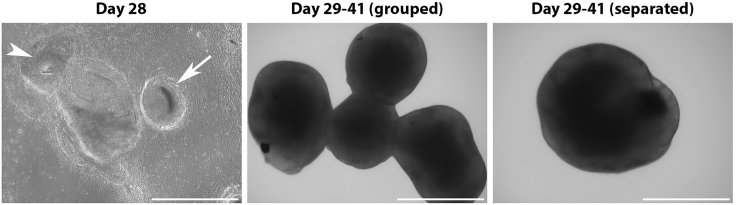
Retinal organoids Derived from Pluripotent Stem Cells A representative bright field image of developing optic vesicle (left, arrow) and undeveloped optic vesicle (left, arrowhead). Representative brightfield images of retinal organoids stuck together (middle) and separated retinal organoid (right). Scale bars=1000 μm.

47. Prepare 3:1 media for dissected organoids: 3:1 media with β-mercaptoethanol (2-ME) at 55 μM final (1:1000 dilution from stock 2-mercaptoethanol at 55 mM purchased from GIBCO), 0.2% IGF-1 (10 μg/mL stock, 1:500), and 2% B27 supplement (1:50).
48. Add 12 mL of media to 100 mm polyHEMA-coated dish.
49. Under a microscope in BSC, dissect optic vesicles identified by their “horseshoe” shape and bright neuroepithelial outer layer with sharp tungsten needles (Figure 2, left, arrow).

50. Use a P1000 pipette fitted to wide-bore tips to transfer dissected optic vesicles to 100 mm dish.
51. Shake plate to distribute optic vesicles evenly in dish.
52. Place 100 mm dish in normoxia incubator and discard 60 mm dish.

### Day 29-41: Half Media Changes with B27 Media

**Timing: 10–15 min**

53. Perform half media changes on Mondays, Wednesdays, and Fridays.
54. Add more media if the culture turns yellow before scheduled media changes, not exceeding 16 mL per plate. Split the optic vesicles into multiple dishes if needed.
55. Under a microscope in BSC, check for optic vesicles sticking together. Separate with tungsten needles and remove poorly differentiated vesicles as necessary (Figure 2, middle).

### Day 42-60: Addition of FBS and Taurine

**Timing: 10–15 min**

56. Prepare 3:1 media with FBS for optic vesicles, by mixing 3:1 +FBS media with 0.1% 2-ME, 0.2% IGF-1, 2% B27 minus Vitamin A supplement, and 1% taurine (100 mM stock, 1:100).
57. Adjust number of plates so that each organoid has ∼1 mL of media. Do not exceed 16 organoids per individual plate. Minimum 12 mL media.
58. Perform half media changes on Mondays, Wednesdays, and Fridays.

### Day 60-90: Addition of 9-*cis* Retinal

**Timing: 20–30 min**

59. Move 100 mm dish to normoxia incubator with front glass door covered in foil.
60. Turn off all lights and allow eyes to adjust to darkness (∼10 min).
61. Under dim red light, prepare 3:1 +FBS media for retinal organoids: 3:1 +FBS with 0.1% 2-ME, 0.2% IGF-1, 2% B27 supplement, 1% taurine, and 1 μM 9-*cis* retinal (10 mM stock, 1:10,000).
62. Adjust number of plates so that each organoid has ∼1.5 mL of media. Do not exceed 10 organoids per individual plate. Minimum 12 mL media.
63. Perform half media changes on Mondays, Wednesdays, and Fridays under dim red light.

**CRITICAL:**

• Minimize all light exposure to prevent isomerization of 9-*cis* retinal to all-*trans* retinal.

### Day 91-Endpoint: Half Media Changes with N2 Media

**Timing: 20–30 min**

64. Under dim red light, prepare 3:1 +FBS media for retinal organoids: 3:1 +FBS with 0.1% 2-ME, 0.2% IGF-1, 1% taurine, 1% N2 supplement (stock, 1:100), and 0.5% μM 9-*cis* retinal (10 mM stock, 1:20,000).
65. Perform half media changes on Mondays, Wednesdays, and Fridays under dim red light.

**CRITICAL:**

• Minimize light exposure to prevent isomerization of 9-*cis* retinal to all-*trans* retinal.

## EXPECTED OUTCOMES

### Accelerated Rod Photoreceptor Differentiation

**Figure 3:**
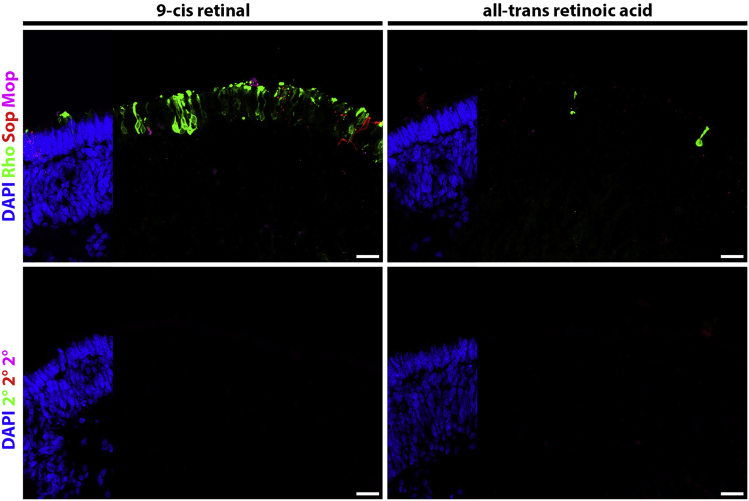
Increased Rhodopsin Expression at Differentiation Day 120 Representative confocal images of the day 120 retinal organoids stained with rhodopsin (Rho, green), S-opsin (Sop, red), and M-opsin (Mop, magenta). Scale bars=10 μm.

Using this protocol, we observe robust Recoverin expression that can be visualized via immunohistochemistry at the apical border of the neural epithelium at day 90. Recoverin expression, in concert with downregulation of *Chx10* expression, indicates that these retinal organoids possess developing photoreceptors (Kaya et al., 2019). Furthermore, robust rhodopsin expression can be visualized in organoids supplemented with 9-*cis* retinal but not in those supplemented with all-*trans* retinoic acid at day 120 (Figure 3).

## LIMITATIONS

The accelerated differentiation protocol for human retinal organoids, presented here, is based on a widely-used protocol in the field (Zhong et al., 2014). Our protocol has been extensively evaluated for at least 9 different human PSC lines. We noted dramatic variations in differentiation efficiency across different cell lines; this variability can be attributed to genetic background of individual lines, passage number, frozen batches, and culture conditions. In addition, though end-point retinal organoids manifest comparable transcriptome profiles, our further analyses indicate down-regulation or shutoff of a substantial number of genes important for phototransduction and/or synaptic formation/function compared to the adult retina, consistent with the view that retinal organoids in culture are not yet functionally mature (Kaya et al., 2019).

## TROUBLESHOOTING

### Problem: Differentiation Efficiency

We note significant variations among human stem cell lines due to genetic and/or experimental factors and some lines fail to generate optic vesicles. The differentiation efficiency can be impacted by cell lines, culture media for PSC maintenance, and experimental procedures. To improve overall efficiency, we refer readers to our recent publication detailing a scraping protocol that produces ∼2.5X more organoids per plate (Regent et al., 2020).

### Potential Solutions

1. Switch to a different cell line from the same donor if one cell line cannot be differentiated after several attempts.
2. Fully characterize the PSC lines by immunostaining of pluripotency markers, karyotyping, and teratoma formation.
3. For induced pluripotent stem cells, we suggest maintaining the same type of culture media when the cell lines are generated from somatic cells.
4. If death of EBs is observed, reduce the number of pipetting.
